# Renewed Urgency: Reimagining Roles in Nursing and Academia Amidst Rapid AI Advancements

**DOI:** 10.3390/ijerph20115963

**Published:** 2023-05-26

**Authors:** Jennie C. De Gagne

**Affiliations:** School of Nursing, Duke University, 307 Trent Drive, DUMC 3322, Durham, NC 27710, USA; jennie.degagne@duke.edu

As a professor of nursing in higher education, I am constantly situated at the nexus of tradition and innovation. The profound transformations brought about by technological advancements are becoming an integral part of our daily lives. Sam Altman’s [1] insightful observation about the “significant impact on jobs” due to artificial intelligence (AI) resonates deeply with the current zeitgeist, prompting us to reflect on how AI’s transformative power can reshape our roles and responsibilities. At the vanguard of this revolution, AI systems such as GPT-4 are emerging not as replacements, but as amplifiers of our human capacities [2]. These tools augment our abilities, facilitating more efficient and effective work. In this context, AI holds promise in areas such as crafting educational content and devising personalized student learning paths, thereby serving as an invaluable ally in our pursuit of knowledge dissemination.

The fear that AI might render jobs redundant is widespread. However, from my vantage point, such fears overlook the unique human elements that are fundamental to both nursing and education. Our professions are deeply rooted in empathy, compassion, interpersonal skills, along with the ability to inspire creativity and intellectual curiosity—elements that current AI technology cannot replicate. Rather than viewing AI as a threat, I perceive it as an opportunity: an innovative tool to enhance our skills, deepen our impact, and enrich our professions.

During this transformative period, open and transparent communication is vital. As a nurse educator, I recognize my responsibility to prepare myself and my students for a future intricately intertwined with AI. This responsibility transcends institutional and national boundaries; it is a global imperative. In our interconnected world, international collaboration among nursing leaders, educators, policymakers, and communities of interest is essential. Such collaboration allows us to collectively shape the future of nursing and academia in the AI era, fostering the exchange of knowledge, experiences, and best practices across borders. 

The promise of AI in nursing and academia, particularly from the perspective of a feminist pedagogy educator, is an exhilarating prospect. By offering a path that embraces deeply embedded principles of equality, empowerment, and social justice, AI has the potential to foster personalized learning experiences. Through accounting for individuals’ unique backgrounds and differences, it encourages an inclusive environment that aligns with the ethos of a feminist pedagogy, enhancing our teaching methodologies and care practices. With AI assuming routine tasks, we find ourselves liberated to focus on providing high-quality, empowering education that cultivates critical thinking, promotes dialogue, and respects diverse perspectives—all key tenets of feminist pedagogy. This newfound capacity can also be harnessed to engage in innovative, intersectional research that addresses systemic disparities within health care and academia. Furthermore, AI aids us in forging deeper, more meaningful connections with our students. It equips us to tailor our approaches according to student needs and preferences, reflecting the emphasis of feminist pedagogy on learner-centered education. Embracing AI provides an exciting opportunity to further the principles of feminist pedagogy and instigate transformative change in nursing and academia.

Throughout my career, I have been guided by the power of human connection in both nursing and education, and AI can assist us in nurturing this connection. I am cautiously optimistic that the integration of AI into our practices can elevate our educational standards, equipping future generations to thrive in a rapidly evolving world. Embracing the AI revolution requires an open and forward-thinking mindset. We must see AI as a tool to enhance our capabilities, not as an end to our professional roles. As we journey through this exciting, yet challenging era, we will remain steadfast in our mission: delivering excellence in education, research, and health care.

As I reflect on the implications and opportunities presented by the AI revolution, I am filled with a sense of urgency. This renewed urgency stems not from fear, but from the recognition that rapid advancements in AI necessitate that we reimagine our roles in nursing and academia. The synergy of our human qualities with these advancements promises a future where we can provide quality education and conduct more effective research. It is an invigorating time to be a part of this transformation, and I eagerly look forward to embracing the opportunities that lie ahead.
